# Methylene Blue Reduces Neuronal Apoptosis and Improves Blood-Brain Barrier Integrity After Traumatic Brain Injury

**DOI:** 10.3389/fneur.2019.01133

**Published:** 2019-11-08

**Authors:** Jun Shen, Wenqiang Xin, Qifeng Li, Yalong Gao, Lili Yuan, Jianning Zhang

**Affiliations:** ^1^Department of Neurosurgery, Yijishan Hospital of Wannan Medical College, Wuhu, China; ^2^Department of Neurosurgery, Tianjin Medical University General Hospital, Tianjin, China; ^3^Tianjin Neurological Institute, Tianjin, China; ^4^Key Laboratory of Post-trauma Neuro-repair and Regeneration in Central Nervous System, Ministry of Education, Tianjin, China; ^5^Department of Neurology, Yijishan Hospital of Wannan Medical College, Wuhu, China

**Keywords:** adenosine triphosphate, apoptosis, blood-brain barrier, methylene blue, reactive oxygen species, traumatic brain injury

## Abstract

**Objective:** To investigate whether methylene blue (MB) treatment can reverse neuronal mitochondrial dysfunction caused by oxygen glucose deprivation/reoxygenation (OGD) injury and then investigate whether MB treatment can reduce neuronal apoptosis and improve blood-brain barrier (BBB) integrity in traumatic brain injury (TBI) animals.

**Methods:** Reactive oxygen species (ROS), mitochondrial membrane potential (MMP), and adenosine triphosphate (ATP) were used to evaluate mitochondrial function. The terminal deoxynucleotidyl transferase-dUTP nick end labeling (TUNEL) assay was used to assess neuronal apoptosis *in vitro*. TUNEL and immunofluorescence staining for neuronal nuclei (NeuN) were combined to assess neuronal apoptosis *in vivo*. An Evans blue (EB) permeability assay and brain water content (BWC) were used to measure BBB permeability *in vivo*. The Morris water maze (MWM), rotarod test, and modified Neurological Severity Score (mNSS) test were employed to assess the prognosis of TBI mice.

**Results:** MB treatment significantly reversed neuronal mitochondrial dysfunction caused by OGD injury. Both *in vitro* and *in vivo*, MB treatment reduced neuronal apoptosis and improved BBB integrity. In TBI animals, treatment with MB not only improved cognitive and motor function caused by TBI but also significantly improved overall neurological function.

**Conclusions:** Our findings suggest that MB is a potential candidate for the treatment of TBI. Future research should focus on other therapeutic effects and mechanisms of MB in secondary brain injury.

## Introduction

Traumatic brain injury (TBI) is the most common cause of mortality and disability among working-age adults and young individuals worldwide (1). In the United States, ~2 million people suffer a TBI each year, and TBI accounts for nearly one-third of all trauma-related mortality (1, 2). TBI damages brain tissue through two pathological processes, primary and secondary injury. Primary injury is characterized by immediate bleeding and loss of brain tissue when a blunt or sharp object impacts the head. Secondary injury involves complicated cellular and biochemical cascade reactions, including oxidative stress, excitotoxicity, neuroinflammation, free radical-induced injury, and calcium-mediated damage, which lead to blood-brain barrier (BBB) damage, elevated intracranial pressure, cerebral hypoxia, brain edema, and neuronal apoptosis (3–8). Mitochondrial dysfunction has been demonstrated to be a key participant in the pathological processes of secondary brain injury (9, 10).

Methylene blue (MB) is an FDA-approved drug used to treat cyanide poisoning, carbon monoxide poisoning, and methemoglobinemia (11). Previous studies have demonstrated that MB can improve mitochondrial function (12). Under pathological conditions, MB acts as an alternative electron carrier that bypasses complex I/III blockage and efficiently transfers electrons from NADH to cytochrome c (cyt c). This process reduces electron leakage, enhances adenosine triphosphate (ATP) production, and decreases the overproduction of reactive oxygen species (ROS) (13). In recent years, MB has been shown to attenuate pathological and neurobehavioral impairments in animal models of Alzheimer's disease (AD) (14, 15), Parkinson's disease (PD) (16), ischemic stroke (17, 18), and TBI (19–21). After TBI, MB treatment can attenuate neuroinflammation, reduce lesion volume, and improve neurological damage (19–21).

Since MB treatment can reduce the release of ROS and increase the production of ATP, it may have the potential to reduce neuronal apoptosis and improve BBB integrity. However, these effects of MB on TBI have not been investigated. In the present study, we first investigated whether MB treatment can reverse neuronal mitochondrial dysfunction and then investigated whether MB treatment can reduce neuronal apoptosis and improve BBB integrity after TBI.

## Materials and Methods

### Normal Cells Culture and Oxygen Glucose Deprivation/Reoxygenation (ODG) Model

PC-12 cells (ATCC, Manassas, VA, USA) and Bend3 cells (ATCC, Manassas, VA, USA) were cultured in Dulbecco's modified Eagle medium (DMEM) (BioInd, Kibbutz Beit Haemek, Israel) supplemented with 100 units/ml streptomycin/penicillin (HyClone, Utah, Logan City, USA) and 10% fetal bovine serum (FBS) (BioInd, Kibbutz Beit Haemek, Israel). Normal cultured cells were maintained in a 5% CO_2_ atmosphere at 37°C. For the OGD model, cells were incubated in glucose-free DMEM and placed in an anaerobic chamber with 5% O_2_, 5% CO_2_, and 80% N_2_ at 37°C for 4 h. A BD Disposable Anaerobic Indicator was used to measure the oxygen level of the anaerobic chamber. After OGD incubation, the glucose-free DMEM was replaced with normal culture medium, and the cells were maintained under normal culture conditions.

### Animals

All animal experimental procedures in this study were approved by the Yijishan hospital and Tianjin Medical University Animal Ethics. Male, weighing 1,822 g (6–8 weeks old), C57BL/6 mice were bought from Experimental Animal Laboratories of the Academy of Military Medical Sciences (Beijing, China). The environment of the animal room was set to 12 h light-dark cycle with a temperature controlled at 20 ± 2°C and humidity controlled at 55 ± 5%. Animals were housed with free access to food and water before experimentation.

### MB Treatment

OGD cells were divided into two groups, the untreated and MB (Jichuan, Taixing, China) treatment groups. In the MB treatment group, the culture medium was treated with 4.5 μM 1% MB (20) at the same time OGD began and was replaced with normal culture medium at the end of OGD. In animals, MB was injected intraperitoneally 1 h after TBI (1 mg/kg), 6 h after TBI (0.5 mg/kg), and at a dosage of 1 mg/kg daily for the next 3 days. The control groups received intraperitoneal injections of the same volume of saline (22).

### Detection of ROS Production

ROS levels were detected using an ROS Detection Kit (BestBio, ShangHai, China). After normal or OGD incubation, the fluorescent probe DCFH-DA (1:2,500) was added to the PC-12 cells, and the cells were incubated for 30 min. The cells were then resuspended after washing and centrifugation, transferred to a flow tube, and analyzed using flow cytometry.

### Mitochondrial Membrane Potential (MMP) Measurement

The MMP of the neurons was measured by a JC-1 Kit (Solarbio, Beijing, China). After normal or OGD incubation, the prepared JC-1 staining working fluid was added to the cells, and the cells were incubated for 20 min at 37°C and then washed with JC-1 staining buffer. Then 2 ml of DMEM was added to each well, and the cells were observed under a fluorescence microscope. The ratio of green fluorescence to red fluorescence was used to represent changes in the MMP.

### ATP Measurement

ATP was measured using an ATP Assay Kit (Beyotime, Shanghai, China). A total of 200 μl of lysate was added to each well of a six-well plate to lyse the cells, and then the cells were centrifuged at 12,000 rpm for 5 min at 4°C. The supernatant was transferred as a specimen for testing. Then 100 μl of ATP test solution was mixed with 10 μl of test specimen or standard specimen, and the optical density (OD) values of the mixture were measured with a spectrophotometer. The ATP concentration was calculated according to the standard curve.

### Establishment of the BBB Model and Permeability Detection

The BBB model was established using a Transwell Kit (diameter 24 mm, pore size 0.4 μm, Corning, NY, USA) according to the manufacturer's instructions. Bend3 cells (1 × 10^6^/ml, 250 μl) were seeded in a travel chamber, and the chamber was transferred to a 12-well plate containing 500 μl of DMEM per well. After incubation for 48 h, the DMEM was renewed, and the cells were incubated for 4 h under normal oxygen or hypoxic conditions. FITC-Dextran (Sigma-Aldrich, St Louis, MO, USA) (250 μl) was added to each chamber, and the cells were incubated for 90 min. Then 100 μl of the solution in the well below the chamber was transferred to a 96-well plate, and the OD (535 nm) values were measured by a spectrophotometer.

### Terminal Deoxynucleotidyl Transferase-dUTP Nick End Labeling (TUNEL) Assay

For cells, the PC-12 cells were plated on glass coverslips after normal or OGD incubation, fixed with 4% paraformaldehyde for 1 h and permeabilized with 0.3% Triton X-100 for 2 min. A total of 50 μl of TUNEL mixture (Roche, Nutley, NJ, USA) was added to each sample, and the cells were incubated for 60 min at 37°C and then incubated with 4',6-diamidino-2-phenylindole (DAPI). For tissue samples, TUNEL and immunofluorescence staining of neuronal nuclei (NeuN) were combined to assess neuronal apoptosis. Frozen sections were prepared 3 days post-TBI and were incubated with an anti-NeuN antibody (1:500, Abcam, Cambridge, MA, USA) at 4°C overnight. After 1 h incubation with Alexa Fluor-conjugated anti-rabbit or anti-mouse IgG (1:500, Thermo Fisher Scientific, Waltham, MA, USA), 50 μl of TUNEL mixture was added, and the sections were incubated for 1 h at 37°C and then incubated with DAPI. A fluorescence microscope was used to determine the number of apoptotic neuronal cells around the traumatic foci. The neuronal apoptosis ratio was recorded for statistical analysis.

### TBI Model

A fluid percussion injury device (Model 01-B, New Sun, Health Products, Cedar Bluff, USA) was used to establish a TBI model. Mice were anesthetized with 10% chloral hydrate by intraperitoneal injection (3 ml/kg) and then placed in a stereotaxic frame. After the scalp was incised sagittally, a 3.5-mm diameter opening was drilled in the right cranium 2.0 mm lateral to the sagittal suture between bregma and lambda. For the sham groups, the surgical procedure was completed. For the experimental groups, a Luer lock connector was placed in the skull opening and cemented in place with cranioplastic cement. The Luer lock was filled with 0.9% normal saline and connected to the fluid percussion device. TBI was induced using controlled fluid percussion as described previously. The incision was sutured immediately, and the animal was placed on a heating pad for recovery from anesthesia.

### Evans Blue (EB) Permeability Assay

For analysis of BBB permeability, mice were injected with 2% EB (3 ml/kg) (Sigma-Aldrich, Tokyo, Japan) *via* the tail vein 72 h after TBI (23). After an hour, the mice were anesthetized with 10% chloral hydrate and then perfused with phosphate buffer saline (PBS) to purge the intravascular EB dye. After decapitation, the ipsilateral hemibrains of the TBI and sham groups were weighed and homogenized in 0.1 g/ml *N,N*-dimethylformamide (Sigma-Aldrich, Tokyo, Japan). Following incubation for 48 h in a 37°C water bath, the hemispheres were centrifuged at 3,000 rpm for 30 min. The supernatants were collected, and the EB absorbance was measured using a spectrophotometer. The concentration was determined from the OD (610 nm) values according to the standard curve, and then the EB content in the brain tissue was calculated.

### Brain Water Content (BWC)

The severity of cerebral edema was evaluated by BWC using the wet weight-dry weight method. Mice were sacrificed 72 h post-TBI, and after anesthesia and decapitation, the brains were removed immediately and divided into the ipsilateral hemisphere, contralateral hemisphere, cerebellum, and brainstem. The fresh tissue samples were weighed immediately to obtain the wet weight and then dried at 100°C for 24 h to obtain the dry weight. The percentage of water content was calculated as follows: brain water content = [(wet weight – dry weight)/wet weight] × 100%.

### Western Blot Analysis

Protein was extracted from injured brain tissues using RIPA buffer, and the concentration was measured by a BCA Protein Assay Kit (Thermo Fisher Scientific, Waltham, MA, USA). Approximately 10 μg of protein per lane was separated using an SDS-PAGE system and transferred to PVDF membranes. The membranes were then blocked with 5% BSA for 1 h at room temperature and incubated with primary antibodies (Caspase 3, 1:1,000, Cell Signaling Technology, Inc., MA, USA; ZO-1, 1:1,000, Cambridge, MA, USA) in 3% BSA at 4°C overnight. After incubation with secondary antibodies (goat anti-mouse or anti-rabbit IgG, 1:5,000, Zsbio, Beijing, China) at room temperature for 1 h, the blots were developed by western lightning chemiluminescence reagents and detected using a Millipore ECL Western Blotting Detection System (Millipore, Billerica, MA, USA).

### Morris Water Maze (MWM)

The MWM test was used to assess cognitive deficits in mice. A circular container (50 cm deep, 150 cm wide) was filled with water at a suitable temperature (22 ± 2°C) and white dye, and a hidden platform was fixed in the center of the container and submerged 1 cm below the water surface. The container was located in a 2 × 2 m room with cues (a square, star, triangle, and circle) on the walls. The data were captured automatically using a video tracking system (Ethovision 3.0; Noldus Information Technology, Wageningen, Netherlands) connected to a computer. All mice were trained for 5 consecutive days after 14 days post-TBI. In spatial learning training, the mice were placed into the container in a random quadrant and allowed to swim in the container until they found the platform. If a mouse failed to find the platform within 60 s, the investigator picked it up and placed it on the platform for 30 s. The platform was removed to evaluate memory retention. The number of crosses into the platform quadrant and the percentage of time spent in the platform quadrant in 60-s intervals were measured.

### Rotarod Test

An accelerating rotarod (RWD Life Science, Shenzhen, China) was used to assess motor coordination and balance. The animals were trained daily for 2 days prior to TBI and were tested 24 h after TBI. Speed gradually increased from 4 rpm to 20 rpm over a 5-min period, and the time that the mice stayed on the rotating cylinder was automatically recorded. The mice underwent three trials with an interval of more than 30 min, and the time spent on the cylinder was averaged over three trials (24).

### The Modified Neurological Severity Score (mNSS)

mNSS measurements were used to evaluate posttraumatic neurological function according to a previous study (24). The test consists of motor (muscle status, abnormal movement), sensory (visual, tactile and proprioceptive), balance beam, and reflex tests. mNSS ranged from 0 (normal function) to 18 (maximal deficit). In the present study, mNSS were assessed on days 1, 3, 5, 9, and 14 days post-TBI. mNSS assessments were carried out by two observers who were blinded to the groups.

### Statistical Analysis

All cellular experiments were repeated at least three times. All experiments were performed in a randomized and blinded manner. SPSS statistical software (version 22.0, IBM) was used for all statistical analyses in the present study. The results are presented as the mean ± SD (standard deviation) and were analyzed using a *t*-test between two groups. One-way ANOVA followed by multiple comparison by LSD test was used for comparisons between multiple groups. The protein band intensity for the Western blots and the fluorescence intensity for MMP measurement were determined using ImageJ software. A *P*-value < 0.05 was statistically significant.

## Results

### MB Treatment Decreases ROS Production Caused by OGD Injury

**Figure 1:**
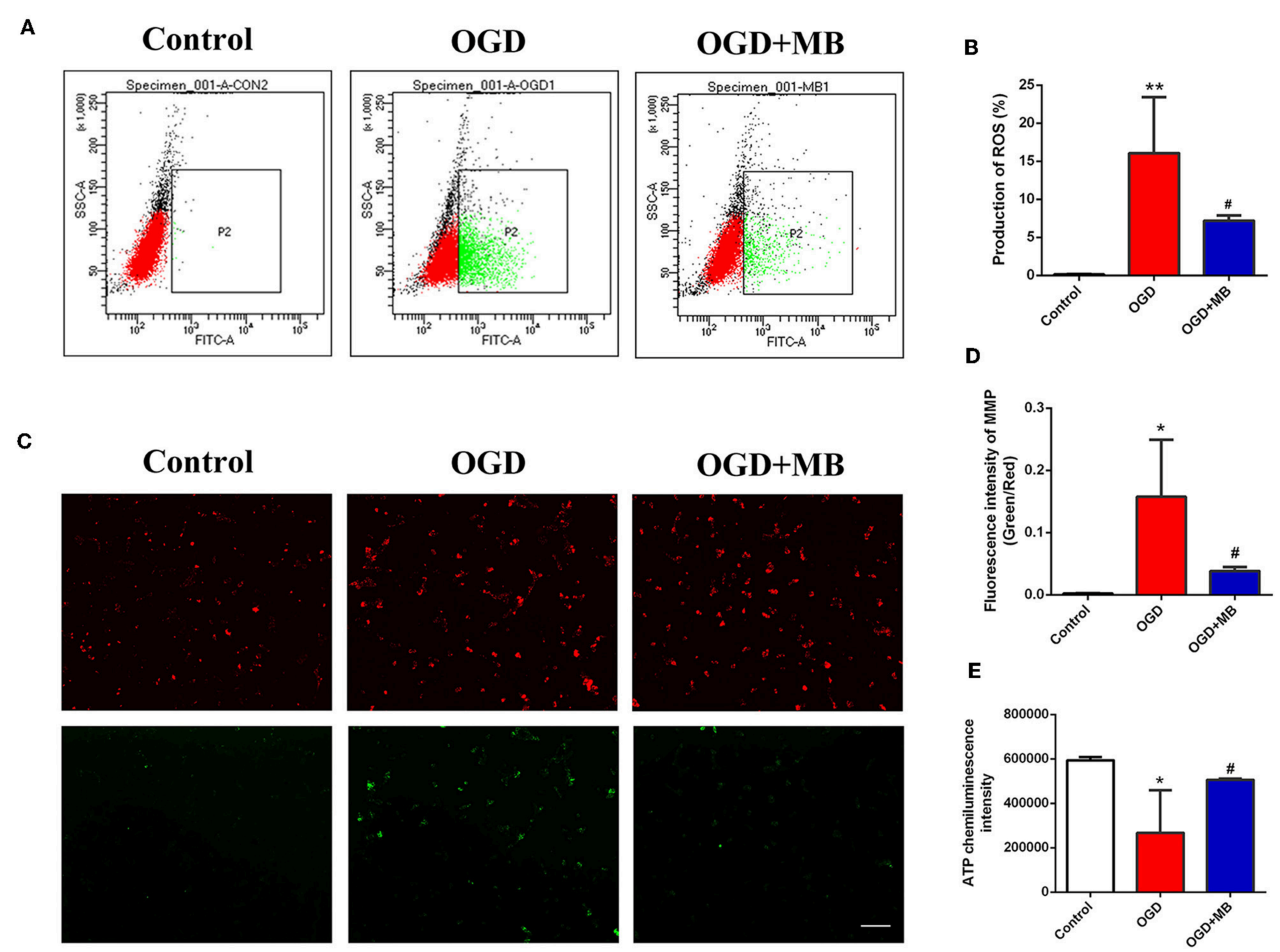
Methylene blue (MB) treatment reversed neuronal mitochondrial dysfunction caused by oxygen glucose deprivation/reoxygenation (OGD) injury. **(A)** Green dot plots represent the level of ROS. **(B)** Quantitative analysis of the reactive oxygen species (ROS) production. ^**^< 0.01, vs. Control group, ^#^< 0.05, vs. OGD group. **(C)** Green fluorescence/red fluorescence ratio represents the MMP stability. Scale bar = 200 μm. **(D)** Quantitative analysis of the MMP, ^*^< 0.05, vs. Control group, ^#^< 0.05, vs. OGD group. **(E)** OD value represents the adenosine triphosphate (ATP) level. ^*^< 0.05, vs. Control group, ^#^< 0.05, vs. OGD group.

After 4 h of OGD incubation, the production of ROS by neurons was significantly increased compared with that of the normal incubation group (*P* = 0.004). The combination of MB treatment and OGD significantly decreased the production of ROS compared with that induced by OGD incubation alone (*P* = 0.044) (Figures 1A,B). This result suggests that MB treatment can significantly reduce neuronal ROS production under OGD injury.

### MB Treatment Stabilizes the Neuronal MMP After OGD Injury

As shown in Figures 1C,D, we used the ratio of green fluorescence signal intensity to red fluorescence signal intensity to represent the stability of the MMP. The stability of the neuronal MMP was significantly reduced in the OGD group compared with the normal incubation group (*P* = 0.011), while MB treatment significantly reversed the decline of MMP stability caused by OGD injury (*P* = 0.033).

### MB Treatment Increases the Production of ATP in Injured Neurons

To determine whether MB treatment can increase the production of ATP in injured neurons, we examined the ATP concentration in the control group, OGD group, and OGD + MB group. As shown in Figure 1E, the ATP concentration was obviously lower in the OGD group than in the control group (*P* = 0.011), and MB treatment significantly decreased ATP consumption compared with that in the OGD group (*P* = 0.039). MB treatment can reduce ROS production, reverse the decline of MMP stability, and increase ATP consumption in neurons under OGD injury. These results demonstrate that MB treatment can reverse the mitochondrial dysfunction caused by OGD injury.

### MB Treatment Decreases Neuronal Apoptosis Caused by OGD Injury

**Figure 2:**
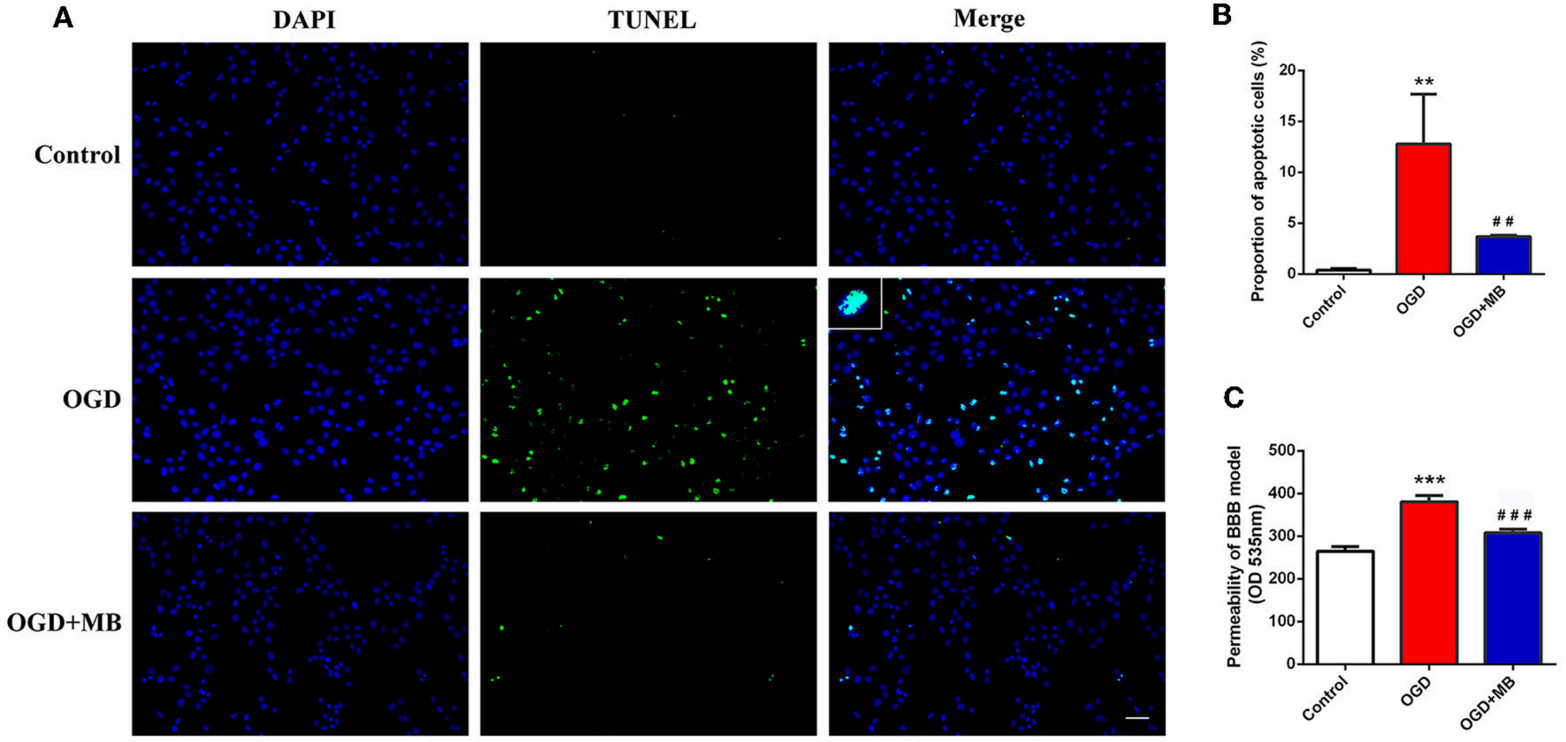
Methylene blue (MB) treatment decreased the neuronal apoptosis and blood-brain barrier (BBB) permeability caused by oxygen glucose deprivation/reoxygenation (OGD) injury. **(A)** Representative fluorescence of apoptotic neurons. Fluorescence colors: terminal deoxynucleotidyl transferase-dUTP nick end labeling (TUNEL): green, DAPI: blue. Scale bar = 200 μm. TUNEL and DAPI double stained cells represented the apoptotic neurons. **(B)** Quantification of apoptotic neurons between the different groups. ^**^< 0.01, vs. Control group, ^*##*^< 0.01, vs. OGD group. **(C)** Quantification of BBB permeability (OD 535 nm). ^***^< 0.001, vs. Control group, ^*###*^< 0.001, vs. OGD group.

Because MB can reverse the mitochondrial dysfunction caused by OGD injury, we speculate that it can reduce neuronal apoptosis after OGD injury. The results showed that the proportion of apoptotic neurons in the OGD group was significantly increased compared with that in the normal cultured group (*P* = 0.002), but the proportion of apoptotic neurons in the OGD with MB treatment group was significantly decreased compared with that in the OGD group (*P* = 0.008) (Figures 2A,B). This result indicates that MB treatment can decrease neuronal apoptosis after OGD injury *in vitro*.

### MB Treatment Can Improve the Integrity of the BBB *in vitro*

A BBB model was established to evaluate whether MB treatment can improve the integrity of the BBB *in vitro*. As shown in Figure 2C, the permeability of the BBB model was significantly increased in the OGD group compared with the control group (*P* < 0.001), and MB treatment significantly improved the integrity of the BBB (*P* < 0.001).

### MB Treatment Decreases Neuronal Apoptosis Caused by TBI *in vivo*

**Figure 3:**
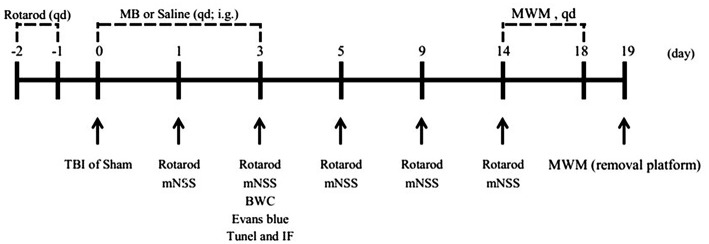
Timeline of the animal experiments.

**Figure 4:**
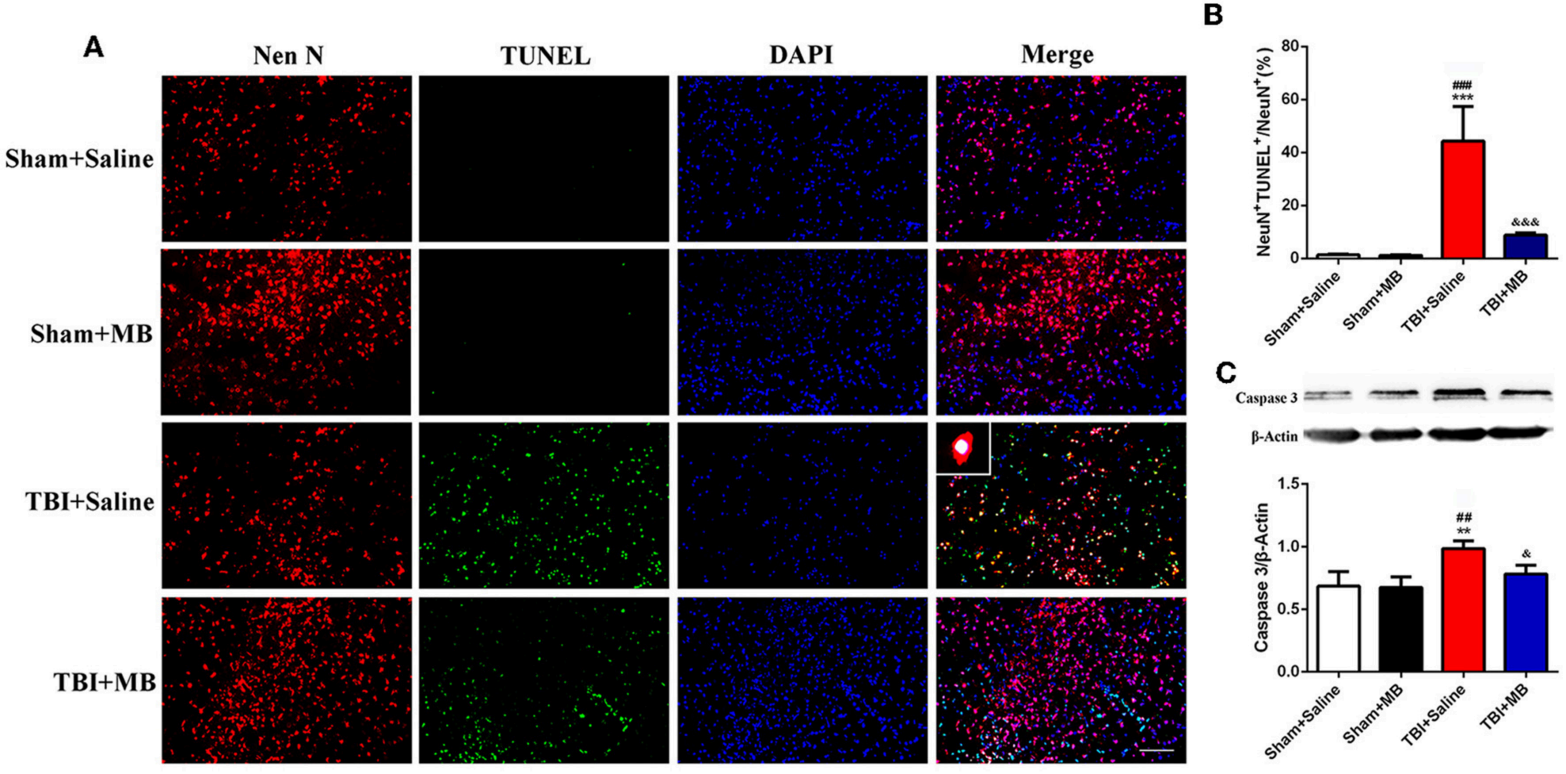
**(A)** Representative fluorescence of apoptotic neurons in the cortex of peri-lesion at 3 days after TBI. Fluorescence colors: NeuN: red, terminal deoxynucleotidyl transferase-dUTP nick end labeling (TUNEL): green, and DAPI: blue. Scale bar = 400 μm. NeuN and TUNEL double stained cells represented the apoptotic neurons. **(B)** Quantification of apoptotic neurons between the different groups. ^***^< 0.001, vs. Sham + Saline group, ^*###*^< 0.001, vs. Sham + MB group, ^&*&&*^< 0.001, vs. TBI + Saline group; **(C)** Representative images and quantitative analysis of caspase 3 expression. ^**^< 0.01, vs. Sham + Saline group, ^*##*^< 0.01, vs. Sham + MB group, ^&^< 0.05, vs. TBI + Saline group.

Since MB treatment can improve mitochondrial function, reduce neuronal apoptosis, and improve BBB permeability after OGD injury *in vitro*, we hypothesized that the administration of MB after TBI can also reduce neuronal apoptosis, improve BBB permeability, and reduce brain edema *in vivo*. To confirm our speculation, neuronal apoptosis, BBB permeability, and brain water content in animal models were assessed. The timeline of the animal experiments is presented in Figure 3. As shown in Figures 4A,B, double staining for NeuN and TUNEL revealed that TUNEL-positive cells were mainly neurons. In the right cerebral cortex of the sham group and the sham + saline group, almost no neuronal apoptosis was observed. In the cortex around the lesion in the TBI group, neuronal apoptosis was significantly increased (vs. the sham + saline group, *P* < 0.001; vs. the sham + MB group, *P* < 0.001), whereas MB treatment promoted neuronal survival (vs. the TBI + saline group, *P* < 0.001). To further confirm that MB can reduce TBI-induced neuronal apoptosis. We examined caspase 3 expression between the groups. The expression of caspase 3 was significantly higher in the TBI + saline group than in the sham + saline group (*P* = 0.003) and sham + MB group (*P* = 0.002), whereas MB treatment significantly reduced caspase 3 expression after TBI (*P* = 0.02) (Figure 4C).

### MB Decreases EB Permeability and Reduces Brain Water Content After TBI

**Figure 5:**
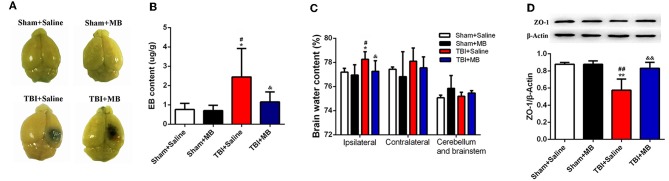
**(A)** Representative images of brain tissues 1 h after Evans blue (EB) injection. **(B)** Quantification of EB content. ^*^< 0.05, vs. Sham + Saline group, ^#^< 0.05, vs. Sham + MB group, ^&^< 0.05, vs. TBI + Saline group, *n* = 46/group. **(C)** Quantitative analysis of brain water content of ipsilateral, contralateral, cerebellum, and brainstem in different groups. ^*^< 0.05, vs. Sham + Saline group, ^#^< 0.05, vs. Sham + MB group, ^&^< 0.05, vs. TBI + Saline group, n = 46/group. **(D)** Representative images and quantitative analysis of ZO-1 expression. ^**^< 0.01, vs. Sham + Saline group, ^*##*^< 0.01, vs. Sham + MB group, ^&&^< 0.01, vs. TBI + Saline group.

To determine whether MB treatment can also improve the decreased integrity of BBB caused by TBI *in vivo*, both EB permeability and brain water content were evaluated. Three days after TBI, the EB permeability in the TBI + saline group was significantly increased compared with that in the sham + saline group (*P* = 0.013) and the sham + MB group (*P* = 0.011). In the TBI + MB group, the EB permeability was significantly lower than that in the TBI + saline group (*P* = 0.047) (Figures 5A,B). The water content of brain tissue from the contralateral side and of the cerebellum and brainstem did not differ between the groups. The water content of brain tissue from the ipsilateral side was significantly higher in the TBI + saline group than that in the sham + saline group (*P* = 0.048), sham + MB group (*P* = 0.013), and TBI + MB group (*P* = 0.041) (Figure 5C). These results indicate that MB treatment can also decrease the BBB permeability caused by TBI. ROS increases the permeability of the BBB by downregulating the expression of the tight junction protein ZO-1 after TBI (4); therefore, ZO-1 expression in the different groups was detected. As indicated in Figure 5D, ZO-1 expression was significantly decreased after TBI (*P* = 0.001 compared with the sham + saline group and *P* = 0.001 compared with the sham + MB group), whereas MB treatment prevented the decrease in ZO-1 (*P* = 0.004).

### MB Treatment Attenuates Neurological Deficits Caused by TBI

**Figure 6:**
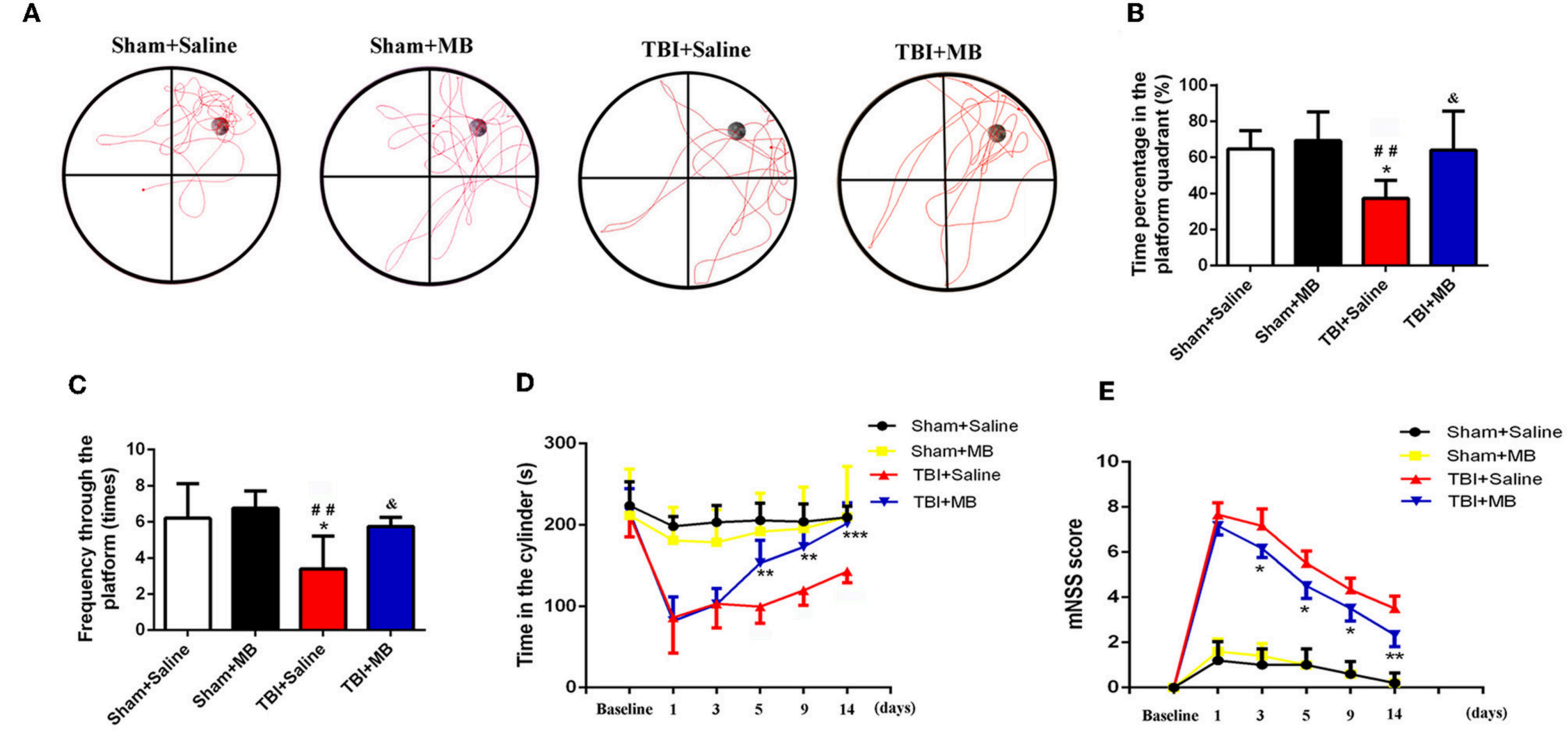
**(A)** Representative images of Morris water maze in different groups, *n* = 46/group. **(B)** Time percentage of the mice in the platform quadrant between different groups. ^*^< 0.05, vs. Sham + Saline group, ^*##*^< 0.01, vs. Sham + MB group, ^&^< 0.05, vs. TBI + Saline group; **(C)** Frequency through the platform of the mice in the different groups. ^*^< 0.05, vs. Sham + Saline group, ^*##*^< 0.01, vs. Sham + MB group, ^&^< 0.05, vs. TBI + Saline group; **(D)** The duration time in the cylinder in the different groups at different time points. ^**^< 0.01, vs. TBI + Saline group, ^***^< 0.001, vs. TBI + Saline group, *n* = 46/group; **(E)** mNSS Score of mice in the different groups at different time points. ^*^< 0.05, vs. TBI + Saline group, ^**^< 0.01, vs. TBI + Saline group, *n* = 46/group.

The restoration of spatial memory was evaluated by the number of crosses into the platform quadrant and the percentage of time spent in the platform quadrant in 60-s intervals. In the TBI + saline group, the time percentage of time spent in the platform quadrant was 37.29% ± 9.99%, which was significantly lower than that spent by the sham + saline group (64.59% ± 10.30%) (*P* = 0.01), the Sham + MB group (69.22% ± 15.86%) (*P* = 0.006), and the TBI + saline group (64.06% ± 21.55%) (*P* = 0.016). The results of the number of crosses into the platform quadrant were consistent with those of the percentage of time spent in the platform quadrant. In the TBI + saline group, the number of crosses into the platform quadrant was 3.40 ± 1.82, which was significantly lower than that of sham + saline group (6.20 ± 1.92) (*P* = 0.011), the sham + MB group (6.75 ± 0.96) (*P* = 0.005), and the TBI + saline group (5.75 ± 0.90) (*P* = 0.035). However, there was no significant difference in either the percentage of time spent in the platform quadrant or the number of crosses into the platform quadrant between the sham + saline group, sham + MB group, and TBI + MB group (Figures 6A–C). These results demonstrate that MB treatment can improve TBI-induced cognitive deficits.

Motor dysfunction and recovery were assessed using the rotarod test. Before TBI induction, rotarod test performance was not significantly different between the TBI + saline group and TBI + MB group (*P* = 0.832). One day and 3 days after TBI, no significant difference in the time spent on the cylinder between the TBI + saline group and the TBI + MB group was observed (*P* = 0.851 and *P* = 0.964). Five days, 9 days, and 14 days after TBI, the time spent on the cylinder by the TBI + saline group was significantly lower than that spent by the TBI + MB group (*P* = 0.004, *P* = 0.002, and *P* < 0.001, respectively). These results suggest that MB treatment can improve the motor dysfunction caused by TBI (Figure 6D).

As depicted in Figure 6E, the mNSS were significantly increased after TBI. On the third day, the neurological function of the mice in the TBI + MB treatment group gradually recovered, and the mNSS were significantly lower than those of the TBI + saline group on days 3 (*P* = 0.017), 5 (*P* = 0.01), 9 (*P* = 0.022), and 14 (*P* = 0.004).

## Discussion

The cascades involved in secondary brain damage following TBI mainly occur in the mitochondria, the dysfunction of which mediates Ca^2+^ overload, cellular excitotoxicity, the release of ROS, and cell apoptosis (9). In addition, the processes of oxidative phosphorylation and ATP generation mainly occur in the mitochondria, which provide ~95% of ATP (25). Once mitochondria become dysfunctional, the production of energy needed for the repair of damaged cells is reduced; this may aggravate cell damage. Therefore, mitochondrial dysfunction plays a pivotal role in the pathological processes of secondary brain damage following TBI, and mitochondria-targeted treatment of TBI may have the potential to improve the prognosis of TBI. Zhu et al. demonstrated that TBI animals treated with SS-31, a mitochondria-targeted peptide, exhibited obviously improved mitochondrial function and reduced secondary brain injury (25).

In the present study, we first detected the effect of MB on mitochondrial function *in vitro* using an OGD model in PC-12 cells. The results revealed that MB treatment can reduce neuronal ROS production, stabilize the neuronal MMP, and increase ATP production, suggesting that MB treatment can reverse the mitochondrial dysfunction caused by OGD injury. However, we did not determine mitochondrial function in neurons in tissue samples from the TBI + saline group and the TBI + MB group because it is difficult to distinguish mitochondria from neurons and from other cells in injured brain tissue.

Second, we demonstrated that the administration of MB in TBI mice can reduce neuronal apoptosis and improve BBB integrity both *in vitro* and *in vivo*. Mitochondria play a pivotal role in neuronal apoptosis after TBI. Under ischemia and hypoxia injury, cytochrome c is released from the mitochondrial membrane and binds with ATP and apoptotic protease activating factor (Apaf-1) to generate apoptosome complexes, which cleave procaspases to activate caspase 3 and induce neuronal apoptosis (26). In addition, the release of ROS and the reduction in ATP production also contribute to neuronal apoptosis (9, 27). MB can transfer electrons from NADH to cytochrome c and increase the stability of cytochrome c. This process increases ATP generation and reduces cytochrome c release and ROS production (13). Therefore, MB can reduce neuronal apoptosis after TBI.

The BBB is composed of pericytes, astrocytes, endothelial cells, and tight junction proteins and is surrounded by neurons (28, 29). After TBI, the release of ROS directly downregulates the expression of tight junction proteins, such as ZO-1 (4). On the other hand, astrocytes transfer their mitochondria to damaged neurons to rescue them (30). These processes directly lead to an increase in the permeability of the BBB. However, neuronal apoptosis also participates in BBB damage. MB treatment can reverse these processes and thus maintain BBB integrity. Moreover, Fenn and his colleagues (20) demonstrated that MB treatment has an anti-inflammatory effect in TBI and that MB treatment directly increases the expression of IL-10 and reduces IL-1β expression in microglia, which can also attenuate inflammatory-mediated BBB damage.

In TBI animals, treatment with MB not only improved cognitive and motor function caused by TBI but also significantly improved overall neurological function. This result was very similar to that of previous studies. Talley Watts et al. (19, 21) showed that MB can minimize neuronal degeneration, behavioral deficits, and lesion volume in TBI animals. Zhao et al. (22) demonstrated that MB exerts a neuroprotective effect in TBI by inhibiting microglial activation, decreasing brain edema, and increasing autophagy. However, while these studies focused on the anti-inflammatory effects of MB in TBI, they did not focus on BBB integrity and neuronal apoptosis, making them quite different from our research. Based on previous studies, MB has three main functions in TBI, namely, exerting anti-inflammatory effects, improving BBB integrity, and reducing neuronal apoptosis, indicating that MB is a potential drug for improving the prognosis of TBI.

Mitochondrial dysfunction following TBI is also involved in many other secondary damages. Synaptic mitochondria are essential for maintaining synaptic plasticity and normal neurotransmission, which dysfunction may lead to neurodegeneration. While synaptic mitochondria have been shown to suffer more injury than non-synaptic mitochondria in a TBI model (31). This reveals that MB improves the spatial memory of TBI mice perhaps partly by improving synaptic mitochondrial function. Additionally, mitochondria can also crosstalk with miRNAs involved in cellular cascade responses to TBI (32), indicating that MB may regulate microRNAs in TBI.

Repurposing old drugs is recommended by modern pharmacology. For example, MB has been used in clinical practice for nearly 130 years, and its safety and side effects have been well-established. Compared with developing new drugs for TBI therapy, repurposing MB has the advantages of saving money and time, and it can be rapidly applied clinically (33). However, although MB can improve the prognosis of TBI mice, the existing research results are not enough to support the immediate use of MB in TBI patients. The other therapeutic effects of MB in secondary brain injury still require investigation in the future.

## Conclusions

This study provides evidence that MB can reverse neuronal mitochondrial dysfunction caused by OGD injury. Both *in vitro* and *in vivo*, MB treatment can reduce neuronal apoptosis and improve BBB integrity. In TBI animals, treatment with MB not only improves cognitive and motor function caused by TBI but also significantly improves overall neurological function. Our findings suggest that MB is a potential candidate for the treatment of TBI. Future research should focus on other therapeutic effects and mechanisms of MB in secondary brain injury.

## Data Availability Statement

All data that support the results of this study are available from the first author upon request.

## Ethics Statement

All animal experimental procedures in this study were approved by the Tianjin Medical University Animal Ethics Committee.

## Author Contributions

JZ and LY designed the study. JS, QL, YG, and WX carried out the experiments. JS, QL, and YG interpreted the results, carried out statistics analysis, and prepared the figures. JS prepared the manuscript. JZ supervised the study and revised the manuscript. All the coauthors listed approved the manuscript.

### Conflict of Interest

The authors declare that the research was conducted in the absence of any commercial or financial relationships that could be construed as a potential conflict of interest.
